# Supplementary material for: “My Pronouns Are”: Pronoun-face mismatch performance and self-report attitudes to gender categorizaton across generations
Source: PLoS One. 2026 Apr 29;21(4):e0343243. doi: 10.1371/journal.pone.0343243 (PMC13127905; doi:10.1371/journal.pone.0343243)
Supplement: S1 Appendix — This appendix contains all supplementary tables and the supplementary figure referenced in the manuscript. (DOCX) [file pone.0343243.s001.docx]

S1 Appendix

Hypothesis One – hypothesised pattern of data

Pronoun-Face


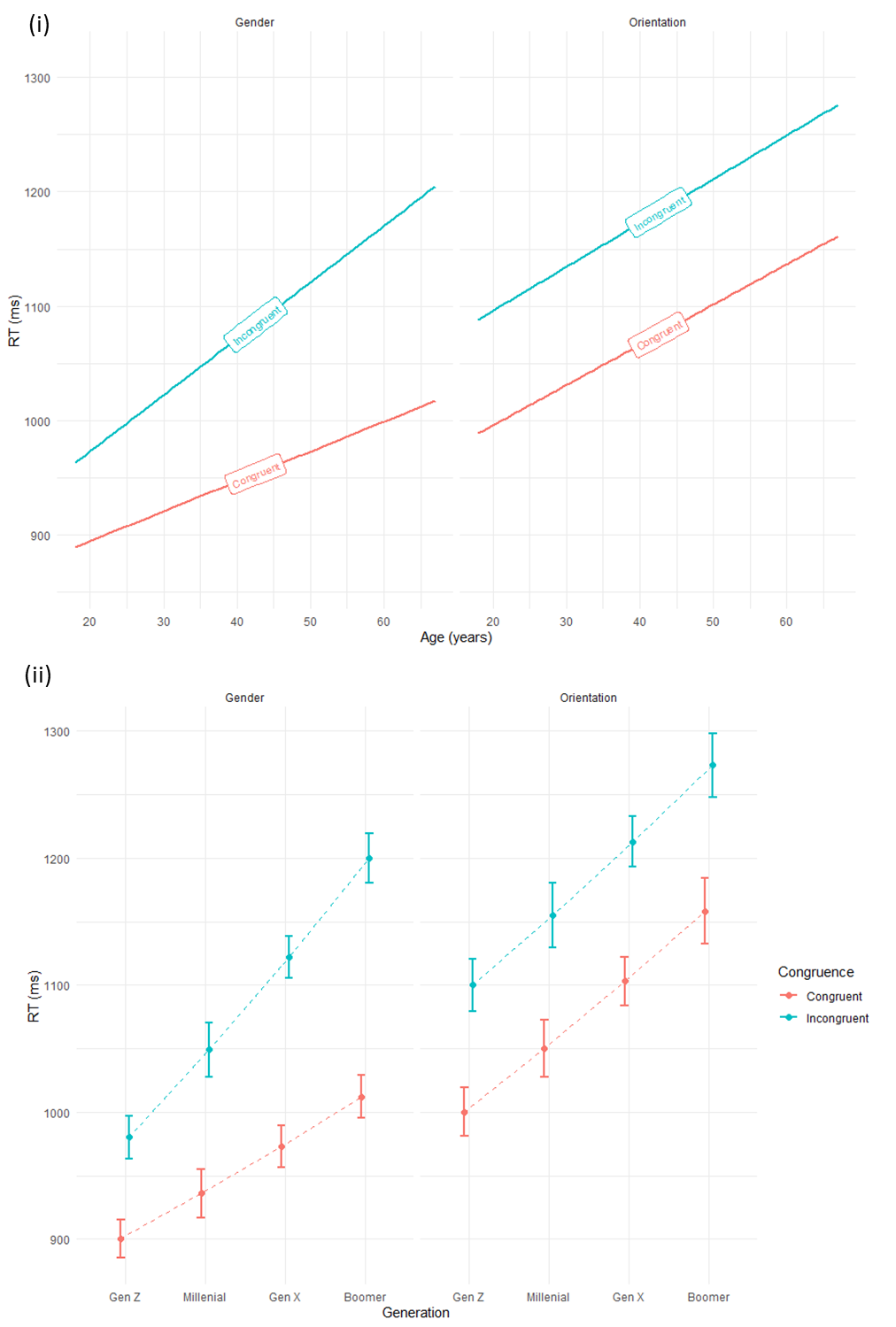


Pronoun-Face

S1 Fig

Hypothesis 1, we predicted that older participants would show greater interference on the pronoun-face and orientation tasks as measured by greater reaction times for incongruent versus congruent trials, and that this effect would be heightened for a pronoun-face task versus a face orientation task. We intended to examine these effects with (i) age in years as a continuous predictor, and (ii) generation as a categorical predictor.

Initial pilot study

One hundred and three English-speaking UK based adults aged 18-67 participated in the study. We excluded five participants due to < 70% accuracy on either the orientation or face-pronoun task. The final sample comprised of 47 self-identified males and 51 self-identified females. Male and female gender was approximately equally represented; none of our participants identified as non-binary or gender-fluid.

**Study Procedure**

The study procedure was the same as reported for our main study except for the following:

- Participants completed study questions prior to completing the tasks rather than after
- Key assignment for the tasks was not randomised
- Participants completed one block of 48 trials per task rather than two
- The study procedure was administered online using Pavlovia and the tasks/questionnaires were created using PsychoPy
- No attention checks were included

**Does performance on the pronoun-face task vary by age?**

We investigated whether older participants performed differently on the tasks using a mixed-effects linear regression model. We used mean reaction times (ms) as a continuous outcome, and age in years, task (Orientation, pronoun-face) and congruency (Congruent, Incongruent), as well as interaction terms between these, as predictors.

Older participants were on average slower (*b* = 4.43, 95% CI: 1.91, 6.95, p = 0.001) but this did not significantly vary by congruency (*b* = 1.20, 95% CI:-0.68, 3.08, p = 0.213), task (*b* = -1.78, 95% CI: -3.66, 0.10, p = 0.065), or age x congruency x task (*b* = -1.16, 95% CI: -3.82, 1.50, p = 0.394).

S1 Table

*Results from a mixed-effects linear regression predicting mean reaction times (ms) from age (years), task, and congruency*

|  | *b* | 95% CI | *p* |
| --- | --- | --- | --- |
| Intercept | 906.31 | 793.87, 1018.76 | < .001 |
| Congruency |  |  |  |
| Congruent (Reference) |  |  |  |
| Incongruent | 84.40 | 0.61, 168.19 | 0.049 |
| Task |  |  |  |
| Orientation (Reference) |  |  |  |
| Pronoun-Face | -6.22 | -124.72, 112.28 | 0.401 |
| Age | 4.43 | 1.91, 6.95 | 0.001 |
| Congruency x Condition | -6.22 | -124.72, 112.28 | 0.918 |
| Congruency x Age | 1.20 | -0.68, 3.08 | 0.213 |
| Condition x Age | -1.78 | -3.66, 0.10 | 0.065 |
| Congruency x Condition x Age | -1.16 | -3.82, 1.50 | 0.394 |

**Do self-reported attitudes to gender pronouns vary by age/generation?**

Four questions were devised to probe for individual self-perception of pronoun use and understanding.

1. How important do you think that it is to use the gender pronoun that an individual identifies by? Possible Responses: Very Easy, Easy, Neither Easy nor Hard, hard, Very Hard.
   1. Collapsed for analysis into: Hard (Very Hard, Hard) vs. Neutral/Easy (Neither easy nor hard, Easy, Very Easy).
2. How easy do you find it to use a gender pronoun that an individual identifies by if they visually appear different? Possible Responses: Very Easy, Easy, Neither Easy nor Hard, hard, Very Hard.
   1. Collapsed for analysis into: Hard (Very Hard, Hard) vs. Neutral/Easy (Neither easy nor hard, Easy, Very Easy).
3. How easy do you find it to use non-binary gender pronouns (they/them) when addressing an individual? Possible Responses: Very important, Important, Neither important nor not important, Unimportant, Not Unimportant.
   1. Collapsed for analysis into: Important (Very Important, Important) vs. Neutral/Unimportant (Neither important nor not important, Unimportant, Not Unimportant).
4. How anxious would you feel if you got someone's pronoun wrong when addressing them? Possible Responses: Severely anxious, Moderately anxious, Mildly anxious, Almost no anxiety, Neutral.
   1. Collapsed for analysis into: High Anxiety (Severely Anxious, Moderately Anxious) vs. Mild/No Anxiety (Mildly Anxious, Almost no anxiety, Neutral).

We investigated whether responses differed by age by conducting separate logistic regression models for each question using the binary variables as described above as outcomes, and age in years as a continuous predictor.

There was a significant difference in self-perception of difficulty in pronoun use by age. For every 1 year increase in age participants became 8.1% more likely to report that using a pronoun that differed from an individual’s visual presentation was ‘hard’ or ‘very hard’ (OR: 1.08, 95% CI: 1.05, 1.12, p < .001). We also found some weaker evidence of a difference in self-perceived ease of using a non-binary pronoun by age. For every one year increase in age participants were 2.9% more likely to report that using non-binary gender pronouns was ‘hard’ or ‘very hard’ (OR: 1.03, 95% CI: 1.05, 1.12, p = 0.041). Furthermore, we found some evidence of a difference in self-perceived importance of using gender pronouns by age. For every one year increase in age participants were 3.1% less likely to report that using gender pronouns that an individual identifies by was ‘important’ or ‘very important’ (OR: 0.97, 95% CI: 0.94, 1.00, p = 0.035). However, we did not find evidence of a difference in self-reported anxiety about using pronouns by age (OR: 1.00, 95% CI: 0.97, 1.02, p = 0.762).

**What is the relationship between self-reported attitudes and performance on the pronoun-face task?**

To examine whether pronoun-face task performance was predicted by response categories for the four questions examining participants’ attitudes to gender pronoun assignment, we conducted a series of linear mixed effects models with interference effects on the pronoun-face task as the outcome. We did not find evidence that self-reported attitudes were associated with performance on the pronoun-face task (S2 Table).

S2 Table

*Results from linear regression models predicting interference effects (difference in reaction times (ms) between incongruent and congruent trials) in the pronoun–face task from self-reported attitudes toward gender pronouns in interaction with age (years)*

|  | *b* | Lower CI | Upper CI | p-value |
| --- | --- | --- | --- | --- |
| *How easy do you find it to use a gender pronoun that an individual identifies by if they visually appear different?* | | | | |
| Intercept | 108.91 | 37.49 | 180.34 | 0.003 |
| Response | -103.31 | -267.00 | 60.37 | 0.213 |
| Age | -0.90 | -2.76 | 0.95 | 0.336 |
| Response * Age | 2.44 | -0.92 | 5.79 | 0.153 |
| *How easy do you find it to use non-binary gender pronouns (they/them) when addressing an individual?* | | | | |
| Intercept | 107.61 | 34.45 | 180.77 | 0.004 |
| Response | -70.88 | -197.29 | 55.52 | 0.268 |
| Age | -0.90 | -2.64 | 0.84 | 0.309 |
| Response * Age | 2.06 | -0.71 | 4.83 | 0.142 |
| *How important do you think that it is to use the gender pronoun that an individual identifies by?* | | | | |
| Intercept | 90.45 | 18.30 | 162.59 | 0.015 |
| Response | -30.70 | -163.54 | 102.14 | 0.647 |
| Age | -0.11 | -1.71 | 1.49 | 0.888 |
| Response * Age | 0.31 | -2.74 | 3.36 | 0.841 |
| *How anxious would you feel if you got someone's pronoun wrong when addressing them?* | | | | |
| Intercept | 69.94 | -10.01 | 149.89 | 0.086 |
| Response | 18.75 | -101.89 | 139.39 | 0.758 |
| Age | 0.18 | -1.60 | 1.97 | 0.841 |
| Response * Age | -0.32 | -3.03 | 2.40 | 0.818 |

Developing Explicit Attitudes to Gender Pronouns Questionnaire

*Initial Generation of Items*

We generated an initial set of 177 items centered around 17 themes for the questionnaire. These items were created through a combination of a literature review on research on gender pronouns, participants sharing their experiences and thoughts of gender pronouns, and prompting ChatGPT to generate items. As a research team we discussed the items, removing those that measured overlapping concepts or those beyond the remit of the questionnaire leaving a total of 53 items.

*Review by Academic Experts*

We then assessed the content validity of the questionnaire by consulting with academic experts of gender identity and pronouns. The experts were given the instructions “*To comprehensively capture the various aspects of attitudes towards gender pronouns we currently have a large number of items grouped into the following subscales:, Importance, Difficulties, Anxiety/Worry, Rumination, Emotional Responses to Misgendering, Intentional Misgendering, Gender Categorisation, Social Norms, Biological Determinism. We are looking to streamline the items to focus on those that are essential for measuring explicit attitudes towards gender pronouns. Please read each item of the questionnaire and indicate whether you think that it is (i) not necessary, (ii) useful but not essential, or (iii) essential by selecting the relevant response. A textbox is provided at the end of each subscale for you to share any specific feedback if you would like to do so*”. We retained items that 50% or more experts rated as essential, leaving a total of 33 items.

*Review by Lay Audience*

As a next step we assessed face validity by asking participants recruited via Prolific to complete the questionnaire, rate each items relevance, and answer some open-ended questions. We retained items that 60% or more of participants rated as quite or highly relevant, leaving a total of 28 items. We added 6 new items based on qualitative feedback as to what aspects of attitudes towards gender pronouns was currently missing from the questionnaire, leaving a total of 34 items.

*Factor Analysis*

We recruited two independent sets of participants for exploratory factor analysis (dataset one; n = 320) and confirmatory factor analysis (dataset two; n = 397). In both datasets participants were recruited via Prolific and were aged 18 years and over, fluent in English and located in the United Kingdom.

We initially conducted an exploratory factor analysis using an oblimin rotation with three factors based on the results of a parallel analysis, and removed items that correlated highly (> 0.80), items with low communalities (< 0.40) in combination with relatively low factor loadings (< 0.70), and items that were cross-loading (< 0.10 difference in loadings between factors). We aimed to create a concise questionnaire, so additional items with relatively low factor loadings compared to others within the factor and those perceived as repetitive were also removed. However, whilst the model showed good fit with EFA in dataset one (χ^2^ (33) = 77.19, < .001, RMSA = 0.065, Tucker-Lewis Index = 0.97), model fit was poor in dataset two when CFA was applied (χ^2^ (51) = 1001.80, p < .001; CFI = 0.56, TLI = 0.43, RMSEA = 0.34 (90% CI: 0.31, 0.36, p < .001), SRMR = 0.14).

We therefore conducted additional EFA using dataset one. Following exploration of data including additional removal of four items with poor loadings, we generated a one-factor solution with eight-items. The model showed a good fit to the data using EFA in dataset one, (χ^2^ (20) = 158.34, < .001, RMSA = 0.04, Tucker-Lewis Index = 0.91) and was validated using CFA in dataset two (χ^2^ (20) = 82.9, p < .001, CFI = 0.998, TLI = 0.998, RMSEA = 0.089 (90% CI: 0.070, 0.109, p = 0.001), SRMR = 0.033).

S3 Table

*Results from CFA for a one-factor solution for the Explicit Attitudes to Gender Pronouns Questionnaire*

| **Item** | **Loading** | **p-value** |
| --- | --- | --- |
| I think that traditional gender pronouns are outdated and do not reflect everyone's experiences. | 0.86 | < .001 |
| I feel comfortable changing my language to use someone's gender pronouns. | 0.90 | < .001 |
| I would feel guilty if I used the wrong gender pronouns. | 0.74 | < .001 |
| I believe that men have penises and women have vaginas, and that this defines their gender pronouns. | 0.89 | < .001 |
| I think that using preferred gender pronouns shows respect. | 0.92 | < .001 |
| I think that society is too focused on accommodating gender identities. | 0.88 | < .001 |
| I feel comfortable with others stating their gender pronouns. | 0.89 | < .001 |
| I think that it's important to use the gender pronouns someone prefers, even if they don't match how I think they look. | 0.95 | < .001 |

*Test-Retest Reliability*

We invited a subset of participants from dataset two to complete the questionnaire approximately one week later to assess test-retest reliability. We found evidence of a very strong correlation for the questionnaire sum scores (ICC = 0.95, 95% CI: 0.92, 0.96, p < .001).

*Concurrent Validity*

We invited a subset of participants from the confirmatory factor analysis to complete the Transgender Attitudes and Beliefs scale to assess concurrent validity. We found evidence of a strong correlation between our questionnaire and the Transgender Attitudes and Beliefs Scale r = 0.81 (95% CI: 0.75, 0.85, p < .001).

Main Study: Trial-level Associations between Age and Task Performance

We analysed the relationship between age and task performance at the trial level using linear mixed-effects models. We log-transformed reaction times (ms) to correct a positive skew and used this as the outcome and entered z-scored age as a continuous between-subject predictor, and task (orientation vs. pronoun-face) and congruency (incongruent vs. congruent) as within-subject predictors (effect coded -0.5, +0.5). We entered participant and face stimuli as a random intercept, and congruency by participant as a random slope.

Results were consistent with our main analysis for H1. We again did not find evidence to support our hypothesis of interaction effects between congruency, task, and age (S4 Table).

S4 Table

*Results from a mixed-effects linear regression model predicting trial-level reaction times (ms) from age (years), task, and congruency, with random intercepts for participants and faces and a random slope for congruency by participant.*

| Predictor | *b* | Lower CI | Upper CI | % Change | Lower CI | Upper CI | *p* |
| --- | --- | --- | --- | --- | --- | --- | --- |
| Intercept | 6.95 | 6.94 | 6.97 |  |  |  | <.001 |
| Congruency |  |  |  |  |  |  |  |
| Congruent (Reference) |  |  |  |  |  |  |  |
| Incongruent | 0.10 | 0.09 | 0.10 | 10.13 | 9.21 | 11.06 | <.001 |
| Task |  |  |  |  |  |  |  |
| Orientation (Reference) |  |  |  |  |  |  |  |
| Pronoun-Face | -0.06 | -0.07 | -0.05 | -5.68 | -6.47 | -4.89 | <.001 |
| Age | 0.07 | 0.06 | 0.08 | 7.39 | 6.00 | 8.80 | <.001 |
| Congruency x Task | -0.01 | -0.03 | 0.01 | -1.06 | -2.61 | 0.50 | 0.178 |
| Congruency x Age | 0.00 | -0.01 | 0.00 | -0.23 | -0.66 | 0.19 | 0.282 |
| Task x Age | 0.00 | 0.00 | 0.00 | -0.07 | -0.37 | 0.23 | 0.658 |
| Congruency x Task x Age | 0.00 | -0.01 | 0.00 | -0.27 | -0.87 | 0.33 | 0.374 |

Main Study: Non-Linear Associations between Age and Task Performance

We explored potential non-linear relationships between age and reaction times for Hypothesis one based on visual examination of the data. We used a scaled exponential relationship to examine a gradual increase in reaction times with age which became steeper as participants became older. However, consistent with our main linear models for hypothesis one we did not find evidence of an interaction between congruency, task, and age (S5 Table).

S5 Table

*Results from a mixed-effects linear regression model predicting mean reaction times (ms) from age (scaled exponential years), task, and congruency*

| Predictor | b | Lower CI | Upper CI | p-value |
| --- | --- | --- | --- | --- |
| Intercept | 971.20 | 943.52 | 998.88 | <.001 |
| Congruency | 103.24 | 84.78 | 121.70 | <.001 |
| Task | -47.00 | -65.46 | -28.54 | <.001 |
| Age (Scaled Exponential) | 8.67 | 6.77 | 10.57 | <.001 |
| Congruency * Task | -12.64 | -38.75 | 13.46 | 0.342 |
| Congruency * Age (Scaled Exponential) | 0.62 | -0.65 | 1.88 | 0.339 |
| Task * Age (Scaled Exponential) | -0.58 | -1.84 | 0.69 | 0.373 |
| Congruency * Task * Age (Scaled Exponential) | -0.34 | -2.13 | 1.45 | 0.708 |

Main Study: Generational effects

As stated in our pre-registration we examined whether cohort effects were best modelled using age in years as a continuous predictor or generation as a categorical predictor by conducting separate models for each of the hypotheses outlined below. We evaluated model fit using the Akaike Information Criterion (AIC), Bayesian Information Criterion (BIC), and where appropriate likelihood ratio tests (LRT). We did not find evidence that either provided a better model fit and results were consistent between models. Full results for generation are therefore presented as supplementary below.

S6 Table

*Results from a mixed-effects linear regression model predicting mean reaction times (ms) from generation (Gen Z, Millennial, Gen X, Boomer), task, and congruency*

| Predictor | *b* | Lower CI | Upper CI | *p-value* |
| --- | --- | --- | --- | --- |
| Intercept | 996.95 | 964.29 | 1,029.62 | <.001 |
| Congruency | 107.03 | 85.34 | 128.71 | <.001 |
| Task | -57.10 | -78.78 | -35.42 | <.001 |
| Generation |  |  |  | <.001 |
| Gen Z | Reference |  |  |  |
| Millennial | 31.25 | -15.03 | 77.53 | 0.185 |
| Gen X | 88.85 | 43.10 | 134.60 | <.001 |
| Boomer | 186.92 | 140.64 | 233.20 | <.001 |
| Congruency*Task | -16.67 | -47.34 | 13.99 | 0.286 |
| Congruency*Generation |  |  |  | 0.902 |
| Gen Z | Reference |  |  |  |
| Millennial | -0.60 | -31.32 | 30.12 | 0.969 |
| Gen X | 4.78 | -25.58 | 35.15 | 0.757 |
| Boomer | 9.84 | -20.88 | 40.55 | 0.530 |
| Task*Generation |  |  |  | 0.766 |
| Gen Z | Reference |  |  |  |
| Millennial | 8.29 | -22.43 | 39.01 | 0.597 |
| Gen X | 9.41 | -20.96 | 39.77 | 0.544 |
| Boomer | -4.83 | -35.54 | 25.89 | 0.758 |
| Congruency * Task * Generation |  |  |  | 0.962 |
| Gen Z | Reference |  |  |  |
| Millennial | 6.11 | -37.33 | 49.55 | 0.783 |
| Gen X | -0.42 | -43.36 | 42.53 | 0.985 |
| Boomer | -5.76 | -49.20 | 37.68 | 0.795 |

S7 Table

*AIC and BIC model comparison statistics for mixed-effects models predicting mean reaction times (ms) using age versus generation (with task and congruency as additional predictors)*

| Model | AIC | BIC |
| --- | --- | --- |
| Model 1 - Age | 30,302.61 | 30,360.44 |
| Model 2 - Generation | 30,235.07 | 30,339.17 |
| Note: Likelihood Ratio Test (LRT): p > 0.05 | | |

S8 Table

*Results from linear regression models predicting self-reported attitudes toward gender pronouns (overall attitudes, perceived difficulties, and worries) from generation*

| Predictor | b | Lower CI | Upper CI | p-value |
| --- | --- | --- | --- | --- |
| *Attitudes to Gender Pronouns* |  |  |  |  |
| Intercept | 28.05 | 26.78 | 29.32 | <.001 |
| Generation |  |  |  | <.001 |
| Gen Z | Reference |  |  |  |
| Millennial | -2.86 | -4.74 | -0.97 | 0.003 |
| Gen X | -3.35 | -5.13 | -1.57 | <.001 |
| Boomer | -4.45 | -6.32 | -2.58 | <.001 |
| *Difficulties using Gender Pronouns* |  |  |  |  |
| Intercept | 7.95 | 7.72 | 8.19 | <.001 |
| Generation |  |  |  | 0.001 |
| Gen Z | Reference |  |  |  |
| Millennial | -0.31 | -0.63 | 0.01 | 0.059 |
| Gen X | -0.27 | -0.60 | 0.06 | 0.110 |
| Boomer | -0.63 | -0.94 | -0.31 | <.001 |
| *Worries about using Gender Pronouns* |  |  |  |  |
| Intercept | 9.87 | 9.33 | 10.40 | <.001 |
| Generation |  |  |  | <.001 |
| Gen Z | Reference |  |  |  |
| Millennial | -0.89 | -1.67 | -0.11 | 0.026 |
| Gen X | -1.17 | -1.94 | -0.40 | 0.003 |
| Boomer | -1.78 | -2.56 | -1.00 | <.001 |

S9 Table

*Model comparison statistics (AIC, BIC) for linear regression models predicting self-reported attitudes toward gender pronouns using age versus generation*

| Model | AIC | BIC |
| --- | --- | --- |
| *Attitudes to Gender Pronouns* |  |  |
| Model 1- Age | 4,232.127 | 4,245.318 |
| Model 2 - Generation | 4,237.059 | 4,259.044 |
| *Difficulties using Gender Pronouns* |  |  |
| Model 1- Age | 2,115.245 | 2,128.436 |
| Model 2 - Generation | 2,116.816 | 2,138.801 |
| *Worries about using Gender Pronouns* |  |  |
| Model 1- Age | 3,195.167 | 3,208.358 |
| Model 2 - Generation | 3,198.217 | 3,220.202 |

S10 Table

*Results from ordinal regression models predicting self-reported attitudes toward gender pronouns (overall attitudes, perceived difficulties, and worries) from age (years)*

| Predictor | OR | Lower CI | Upper CI | p-value |
| --- | --- | --- | --- | --- |
| *Attitudes to Gender Pronouns* |  |  |  |  |
| Age | 0.80 | 0.73 | 0.88 | <.001 |
| *Difficulties using Gender Pronouns* |  |  |  |  |
| Age | 0.83 | 0.75 | 0.91 | <.001 |
| *Worries about using Gender Pronouns* |  |  |  |  |
| Age | 0.81 | 0.74 | 0.89 | <.001 |

Note. Age was scaled for interpretability so that coefficients represent the change in odds ratios for a 10-year increase in age.
