# Supplementary material for: “My Pronouns Are”: Pronoun-face mismatch performance and self-report attitudes to gender categorizaton across generations
Source: PLoS One. 2026 Apr 29;21(4):e0343243. doi: 10.1371/journal.pone.0343243 (PMC13127905; doi:10.1371/journal.pone.0343243)
Supplement: Attachment — Submitted filename: Reviewers_Response.docx [file pone.0343243.s003.docx]

Manuscript information:
========================
PONE-D-25-31694
Age-Related Discrepancies in Gender Pronoun Attitudes: a Mismatch between Implicit and Explicit Measures
PLOS ONE
========================

Dear Dr Roberts,

Thank you for the opportunity to revise and resubmit our manuscript, previously titled “Age-Related Discrepancies in Gender Pronoun Attitudes: a Mismatch between Implicit and Explicit Measures” for consideration for publication in PLOS One. In line with Reviewer 1’s comments regarding the framing of the paper, we have now re-titled it “My Pronouns Are”: An Investigation of Pronoun-Face Mismatch and Self-Report Attitudes to Gender Categorizaton Across Generations”. We have changed the name of the task from gender pronoun Stroop task to pronoun-face congruence task to reflect the reviewers’ valid point that our task is not a classic Stroop paradigm. We have also gone through all supplementary materials and figures and worded accordingly. We appreciate the detailed feedback from the reviewers and outline how we have responded to each point below (*in italics*). We feel these revisions have deeply strengthened the paper and thank you and the reviewers for your engagement and helpful suggestions.

**Reviewer #1**: The manuscript addresses a highly interesting and socially relevant topic. The study is innovatively designed, and it is evident that the authors have made a commendable effort to ensure transparency and rigor in terms of materials, data collection, and analysis. However, the current version of the manuscript does not sufficiently explain key concepts, and it remains unclear what the experimental task is intended to measure and to what extent the experimental and explicit measures address overlapping concepts. As a result, the overall contribution of this study remains unclear. Specifically, the manuscript would benefit from a more precise theoretical framing of the experimental task and a clearer explanation of how attitudes are expected to manifest in the response patterns. 
 
Specific comments: 
The term 'gender pronoun attitude' (a key concept in the manuscript) is not clearly defined in the introduction. Furthermore, this concept is referred to inconsistently throughout the text (e.g. 'attitudes towards pronoun assignment' in the abstract, 'explicit attitudes to gender pronoun categorisation' on page 4, and 'self-perception of difficulty using pronouns' on page 5). A more precise and consistent definition is required to clarify the theoretical framework and the study's objectives.

*Response:* *We have defined and been more consistent with our terminology. We now use the term explicit attitudes to preferred gender pronouns and pronoun categorization and define this as beliefs regarding traditional and preferred pronouns. This is explored here using our novel questionnaire which investigates attitude, difficulty and worry using gendered pronouns.*
 
I am not convinced that describing the task as a Stroop task is the best approach. The task appears to be more accurately described as a pronoun–face congruence task, in which participants judge the semantic match or mismatch between a face and a pronoun. That is, they must decide whether the gender association evoked by the face matches that evoked by the pronoun. In a Stroop-like task, I would have expected automatic gender associations to interfere with the processing of another dimension, such as categorising photos presented visually, regardless of the gender of the pronouns presented, where inhibition and control mechanisms would be relevant.

*Response: We thank the reviewer for bringing this to our attention and agree that the paradigm is better described as a pronoun-face congruence task with a match/mismatch response which differs from the classic Stroop paradigm; we have altered the manuscript accordingly throughout. We also further describe the origin of our task in relation to the different auditory tasks (Green & Barber, 1981; Most et al, 2007) and explain why we were looking at congruence as a measure of interference. We have removed discussion of the longitudinal Stroop studies and refer to more recent work looking at interference in general. We also acknowledge that we do not know whether it is familiarity or interference that generates our pronoun-face congruence interference and that it has not been studied longitudinally. We also acknowledge in discussion that the classic Stroop generates interference across another dimension.*

I am also unsure what the task is intended to measure. In terms of attitude, one could argue that the quicker someone can identify congruent trials (e.g. a photo of a woman paired with the word 'she') as matches and incongruent trials (e.g. a photo of a man paired with the word 'she') as mismatches, the more traditional their gender assignments tend to be. As gender-congruent pairs are expected to be more familiar, differences in recognition times between matches and mismatches could reflect processing costs, as familiar combinations are easier to process. However**,** I am unsure how this can be interpreted as an expression of implicit 'attitudes towards gender pronouns'. It would be helpful if the authors would provide a more detailed explanation of the cognitive processes involved in performing the task they designed, along with the rationale behind it.

*Response: We agree with the reviewer’s summation that speeded responses on our pronoun-face congruence task is likely to reflect familiarity (which we have specifically addressed further in the introduction), We have removed all referral to implicit attitudes throughout and simply talk about congruence/incongruence interference. We have further elaborated why we were looking at processing costs based on the Implicit Attitudes Test literature but also include discussion of the controversial interpretation of how it related to explicit attitudes (see same point below).*

The extent to which the experimental task and the explicit measures (questionnaire) address overlapping constructs is unclear. The explicit measure assesses attitudes towards the assignment of pronouns based on biological characteristics versus self-identification. However, it is unclear how a positive attitude towards self-selected pronouns would manifest in the response patterns of the experimental task. For example, if a participant believes that pronouns should not be assigned based on biological or facial features, they might consider a traditionally masculine-looking face paired with 'she' to be an appropriate match. However, such responses appear to have been excluded from the analysis as 'inaccurate'. It would be important to explicitly explain how attitudes are expected to manifest in the response patterns.

*Response: First, we agree with the reviewer that the relationship between reaction time interference tasks and explicit attitudes is not straightforward (as per the controversial over interpretation of the Implicit Attitudes Test that we address in the introduction). We have sought to clarify why one might expect a relationship in the introduction. Specifically, individuals who explicitly expressed difficulty in applying pronouns might have had stronger biases towards typical match/mismatch categorization on a speeded task. However, as in our earlier pilot, this prediction was not supported here, even after improving the explicit measure (now validated) and increasing the sample to address the earlier power concern. We now state this more clearly in our discussion.*

*As to the second point, it is true that if an individual consistently believed that pronoun-face incongruence to be an appropriate match, then they would have been removed from analysis as this would have resulted in low accuracy. Our pre-registered data-quality criterion (< 70% accuracy on ≥1 task) follows standard practice for speeded RT paradigms, where very low accuracy typically indicates that RTs are not interpretable (e.g. guessing, inattention). Twenty-six participants met this preregistered exclusion. As the reviewer notes, it is* possible *that a small subset of participants responded “inaccurately” because they personally endorsed pronoun–face pairings that differed from the experimental rule. We explored this by looking for the pattern one would expect under that interpretation, namely relatively good performance on the orientation task but poor performance on the pronoun-face task. We found only 9 such cases (≈1.3% of participants who completed the study procedure). This number is too small to re-estimate the RT–attitude relationship with adequate power, so we have not conducted a separate analysis. However, to facilitate further work on this question, we have made the full dataset, including excluded cases, openly available so that alternative scoring rules or inclusion criteria can be applied.*

Reviewer #2:

In general, I liked the idea of the study, especially the fact of including the age factor to be analyzed. Still, I do not think that the paradigm is very novel (other authors have used very similar tasks), the literature review is not very actual, and I miss some conditions to deeply explore topics related to alternative gender pronouns and gender fluid representation. As a positive note, I would like to remark on the effort of the authors in collecting a huge amount of data, and on doing a very sophisticated pilot study. This provides great statistical support to the findings, which is laudable.

*Response: We appreciate that the reviewer likes the idea, the age analysis and the great statistical support. We have reframed the paper in line with Reviewer 1’s suggestion above and reworked the literature review accordingly.*

*The study is indeed similar to other older auditory Stroop studies (e.g. Green & Barber (1981) Auditory Stroop. Male v Female Voice speaking “Man” v “Girl” Most et al., (2007) uses gender category words and vocal response “Boy” or “Girl” Looked at children and adults). However, as suggested, we have added the most recent study by Serafini, L., & Pesciarelli, F. (2025) which looked at whether gendered words primed Italian he/she pronouns or male/female faces. We agree that our study cannot deeply explore topics related to alternative gender pronouns gender fluid representation, and acknowledge this in the discussion.*

Below I present chronically my comments and suggestions, which I think will increase the quality of the study.

Pg. 9 line 36 “a uniquely millennial phenomenon which sees many young people rejecting the notion that sex and gender need to be correlated, with some individuals wholly rejecting gender as a binary construct”

– I do not think this is an exclusive millennial phenomenon. Indeed, many cultures have gender fluid members well categorized for centuries. See for instance Hijra (South Asia), Muxe (Oxaca, Mexico), Bugis, Bissu, Calalai (Indonesia), Mahu (Hawaii) among others. This has been a reality for non-western societies, and we should not invalidate them as individuals or talk about it as a fashion for young people. People who do not categorize their gender with their biological sex has always existed and we should acknowledge this reality.

*Response: We thank the reviewer for this important point. We have removed the phrasing ‘uniquely millennial’ and specified this is in relation to Western cultures. We have also now explicitly acknowledged the range and reality of gender fluid cultures at the end of the introduction section.*

Pg. 9 line 39 “coding for sex is automatic and difficult to suppress in adults”

– the reference for this quote is a study about race, not sex. Moreover, the result of the study posits that it is possible to change the perception of race: “an alternate social world was enough to deflate the tendency to categorize by race. ” Therefore, I think that this statement needs to be supported by the literature, or either toned down because despite the coding might be automatic, I am not sure it will be hard to suppress.

*Response: We have removed the reference to race and provided an alternative source related to gender categorization.*

Pg. 9 line 40: cite no. 4 reference a study from 1978. I suggest doing another search in the literature so you could include more recent studies tackling the same topic. For instance,it comes to my mind the study by Van den Brink et al., 2012 with voice processing that is also rather old. For sure, more recent research has been done, please try to find something newer.

*Response: We have included more recent research as requested (eg. Serafini, L., & Pesciarelli, F. 2025, Reference No. 9).*

Pg. 9 line 53: cite no. 6: the study is also a bit old. You can find more recent references and similar studies using the sex of the speaker to trigger incongruence, like for instance the study by Casado et al., 2021

*Response: We have now included the Casado et al. (2021) reference and thank the reviewer for pointing us to this paper. We agree that the other stated citation is a bit old, but have chosen to keep it in the paper as it is the most directly relevant to our paradigm because it looks at voices and pronouns that are congruent and incongruent and complements the Casado, Palma & Paolieri paper.*

Pg. 10 line 59: cite no. 7 from 1990. Include also a reference more contemporary.

*Response: We have included another more recent reference (Pettigrew et al., 2014) that shows interference increases with age.*

Pg 10 line 61: cite no. 8 – I am sure that a more recent metanalysis has been done since

1998, please, do a literature search to actualize the reference.

*Response: In accordance with the request of Reviewer 1, we have removed this meta-analysis and much of the discussion related to Stroop tasks.*

Pg 10 line 63 “As individuals gain experience with certain types of faces, especially those encountered more frequently, facial processing becomes more efficient but also more entrenched”

– this information is rather surprising for the reader. Until now you are talking

about interference and inhibition in general, and suddenly there is a jump to face

processing. I miss a link of information here.

*Response: We have added another example (the other race effect; Hancock & Rhodes, 2008; Reference No. 13) of face processing which displays this entrenchment phenomenon related to experience to draw the link between strength of pronoun-face matching with age.*

Pg. 11 line 81: “In this study…”

– I think your procedure is very similar to the one used by

Serafini & Pesciarelli 2025. Please, have a look at this study and cite them.

*Response: This study is now cited earlier (Reference No. 9).*

Pg. 18 line 222 “Participant was included as random intercept to account for within-

subject variability”

– I think this is the correct decision. However, I wonder why you did not include the faces as random intercept as well, this way, you could also account to the variability of your stimuli and will increase the power of your analyses. I strongly suggest the Authors to include the stimuli (item) intercept, and also, to create the maximal models following Barr et al., 2013 recommendation. In your case, (1 + stroop task + congruency| participants) + (1 + age + stroop task + congruency | items). Aso, make sure before including the continuous variables in the task that they are normally distributed (including RTs), otherwise you may need a transformation. Moreover, specify the contrast selected for the categorical variables—if you do not have specific predictions about the direction, Schad et al. (2020) usually recommend using a sum contrast (-0.5, 0.5)

*Response: Thank you for the suggestion to consider the effect of faces. We did not do this in our original analysis as we modelled data at a mean RT level (i.e., collapsed across trials). We have now addressed this by conducting an additional trial-level analysis which takes into account the effect of faces, in line with the reviewer’s suggestions.*

*As the reviewer suggested, we initially used a maximal model following Barr et al’s recommendations (2013): log_RT ~ age * task * congruency + (1 + task * congruency || participant) + (1 + task * congruency || face). However, this model failed to converge and produced singular fits, indicating that several variance components, particularly the by-face slopes and the by-participant task slope, were effectively zero and not supported by the data. We would like to note that we did not include age as a random effect in this model as age is a between-subject variable and therefore constant within participant and face stimuli.*

*Following this initial model, we retained the simplest non-singular structure supported by the data: log_RT ~ age * task * congruency +(1 + congruency | participant) + (1 | face), in line with current recommendations (Bates et al., 2015; Matuschek et al., 2017). This model included a random intercept for faces, and a by-participant random slope for congruency. This model converged normally and avoided singularity. Adding by-participant or by-face task slopes did not improve model fit and left fixed-effect estimates unchanged.*

*As the reviewer suggested, we used log-transformed reaction times (ms) to correct a positive skew, and effect-coded the categorical predictors (-0.5, +0.5), within these models.*

*Our results were consistent with the mean RT model reported in the manuscript. We have therefore retained the mean RT model as the primary analysis for H1, but report the trial-level model as supplementary (see Supplementary Table 4 for full results).*

*References*

Barr, D. J., Levy, R., Scheepers, C., & Tily, H. J. (2013). Random effects structure for confirmatory hypothesis testing: Keep it maximal. *Journal of memory and language*, *68*(3), 255-278.

Bates, D., Kliegl, R., Vasishth, S., & Baayen, H. (2015). Parsimonious mixed models. *arXiv preprint arXiv:1506.04967*.

Matuschek, H., Kliegl, R., Vasishth, S., Baayen, H., & Bates, D. (2017). Balancing Type I error and power in linear mixed models. *Journal of memory and language*, *94*, 305-315.

Schad, D. J., Vasishth, S., Hohenstein, S., & Kliegl, R. (2020). How to capitalize on a priori contrasts in linear (mixed) models: A tutorial. *Journal of memory and language*, *110*, 104038.

Pg. 18, line 224 “For our second hypothesis, we used a linear regression model with total scores on the Explicit Attitudes to Gender Pronouns questionnaire as a continuous outcome, and age in years as a predictor” – Here also make sure of what is the distribution of your data, and whether it needs to be transformed in order to reach normal distribution

*Response: We assessed normality using Q–Q plots of the model residuals, which showed approximately normal central distributions across scales. As the difficulties and worries scales comprised a small number of items (two and three respectively), the data were discrete and bounded, resulting in minor deviations in the tails. Given the relatively large sample size, we expected the models to be robust to these deviations. However, in light of the reviewer’s comment, we have taken additional steps to address this by re-estimating the models with heteroskedasticity-consistent (HC3) robust standard errors for confidence intervals and p-values. We also conducted proportional-odds ordinal models as sensitivity analyses, which confirmed that the pattern of results (see Supplementary Table 10).*

Pg. 18 line 229 “For our third hypothesis, we calculated the difference in reaction times (ms) between incongruent and congruent trials in the gender Stroop task and used this as a continuous outcome in a linear regression model. We entered age in years and sum scores for each set of questions as predictors independently and in interaction in individual models”

– I understand your reasoning, and I think is correct. Still, I believe you could obtain more information if you include in your first model the sum scores for each set of questions as a factor. In that way, with one model you could explore 2 hypotheses and the control will be higher.

*Response: We appreciate the reviewer’s suggestion and explored a combined model including all three questionnaire scores scaled to a common 0–100 range. We used a linear regression model with each score entered as a separate variable in a wide format, independently and in interaction with age. The results were consistent with the analyses reported in our manuscript (no significant effects). As findings were consistent and we consider the items to reflect distinct constructs we have chosen to retain our pre-registered original models in our manuscript. However, for the reviewer’s interest we have provided the combined model results below.*

| **Predictor** | ***b*** | **Lower CI** | **Upper CI** | **p** |
| --- | --- | --- | --- | --- |
| Intercept | 102.76 | 15.90 | 189.61 | 0.020 |
| Age | -0.36 | -2.21 | 1.48 | 0.700 |
| Attitude to Gender Pronouns | -0.60 | -1.51 | 0.31 | 0.193 |
| Difficulties using Gender Pronouns | -0.18 | -1.36 | 1.00 | 0.762 |
| Worries about using Gender Pronouns | 0.60 | -0.16 | 1.37 | 0.122 |
| Age * Attitude to Gender Pronouns | 0.02 | 0.00 | 0.04 | 0.087 |
| Age * Difficulties using Gender Pronouns | 0.00 | -0.02 | 0.03 | 0.796 |
| Age * Worries about using Gender Pronouns | -0.01 | -0.03 | 0.00 | 0.127 |

Pg. 20 line 253: I miss a table with the model outcomes. Particularly, for me, it is more intuitive to check the values at the table rather than inside the text. I believe that originally you created table 2 but for some reason it is not included in the document.

*Response: We have now included table 2 with the full model results in the manuscript.*

Pg. 20, Figure 1: the color selection seems to be by default, and this makes the graph not very intuitive: usually we associate red with incongruent and green with congruent. Please check how to change the color palette and select a different scale (it is very simple, there are many tutorials online); also remember to change the color of the line and the color of the ribbon as well (for the ribbon you can also modify the transparency). Also, it is possible to modify the grid (the way it is presented now is overwhelming) – check different “theme” options.

*Response: We have amended the line colours and confidence intervals in Figure 1 so that green is now congruent and red is incongruent. The grid issues were caused by our method of exporting the file leading the background to be transparent. We have altered this so the background is white which will make the figure clearer in Plos ONE’s reviewer system.*

Pg. 22, table 3: maybe you could accompany the table with a small graph --https://www.sthda.com/english/wiki/correlation-analyses-in-r here you can chose the preferred visualization

*Response: We thank the reviewer for suggesting that we provide a visual accompaniment to Table 3. Table 3 presents regression coefficients rather than zero-order correlations, so a correlogram would not be appropriate. However, in line with the reviewer’s suggestion, we have added a figure displaying the estimated effects (with 95% confidence intervals) for these models.*

Pg 27, line 332 “All participants had quicker responses in the gender task compared to the

control (orientation) condition, suggesting that responses for orientation are more

automatic and thus more difficult to inhibit. ”

– in my opinion, this result also can be interpreted such that stereotypes do not evoke such a greater interference effect as the more perceptual task with face orientation, such as the stereotypical activation is less automatic. What you mention in line 339 also aligns with this idea “it is possible that our task required less conceptual level inhibitory control than classic colour interference tests”

*Response: We have added this alternative interpretation (lines 380-381).*

.

Pg. 28 line 345 “This is consistent with literature characterising younger adults as being

socially conscious about gender identity [22,23], and present with increased self awareness and acceptance of people and wider societal concerns [24,25]. ”

here you should specify “younger adults from western societies”

*Response: This has been added (line 395).*

.

– I think that

Pg. 28 line 348 “Older generations have more exposure to traditional pronouns”

– Here I miss the explanation of what is considered a traditional pronoun in opposition of what is considered a non-traditional pronoun and examples. Also, specify that older generation of western societies.

*Response: This has been added (lines 38-41).*

Pg. 28 line 353 “This study is the first to demonstrate two key results: binary pronoun

assignment is an automatic process, and it is not processed differently at an implicit level

by adult age”

– I do not think you can arrive to this conclusion based on your data; in order

to give such an statement, you should have compared the non-binary pronoun

assignment. You could say that this study demonstrates that binary pronouns trigger an

automatic categorization processing based on biological sex distinction, and that you are able to observe this in relation on how the pronouns are associated to normative gender faces. Keep in mind that in your study you did not include alternative pronouns such as “they” neither alternative faces, such as androgyne looking people, transgender people, or non-binary people.

*Response: We agree with the reviewer and have amended the conclusion accordingly (lines 406-408).*

Pg 28 line 355 “This aligns with literature on automatic categorisation of gender [26]”

paper is from 1984; I am sure that since then, Sex Roles published more recent studies

exploring the automatic categorization of gender.

*Response: We now cite a 2025 study that demonstrates automatic categorization of face into gender (reference 32).*

Pg. 28 line 357: “Rekow and colleages…”

– here you should also discuss your results in comparison with that of the study by Serafini and Pesciarelli 2025 that I mentioned before(they also included EEG measures).

*Response: We have now included a longer discussion of Serafini and Pesciarelli 2025 (lines 412-417).*
