# Supplementary material for: “My Pronouns Are”: Pronoun-face mismatch performance and self-report attitudes to gender categorizaton across generations
Source: PLoS One. 2026 Apr 29;21(4):e0343243. doi: 10.1371/journal.pone.0343243 (PMC13127905; doi:10.1371/journal.pone.0343243)
Supplement: Attachment — Submitted filename: Reviewers_Response_auresp_2.docx [file pone.0343243.s004.docx]

Reviewer #1: Overall, the manuscript shows significant improvement and much greater clarity. Most of my previous comments have been satisfactorily addressed. One issue remains unresolved, and I have a few additional comments for consideration. Thank you for your careful revisions and for engaging thoughtfully with the feedback—I appreciate the effort invested in strengthening the manuscript.

*Response: We thank the reviewer for their careful reading of the revised manuscript and for the constructive feedback throughout the review process. We appreciate the detailed engagement with our work and the acknowledgement of the improvements made. We address the remaining issue and all additional comments in full below and have made the corresponding revisions in the manuscript.*

There remains a lack of conceptual clarity regarding the self-report measure. In the supplementary materials, the questionnaire is presented as “Attitudes towards gender pronouns” with content validity checked by academic experts. Including the instructions provided to these experts in the main text might help clarify the underlying concept(s).

*Response: We are currently drafting a research paper fully detailing the construction and psychometric validation of the self-report measure we have used in this study. We believe the full methods and results of this process would be beyond the remit of the current research paper. However, we have detailed the process briefly in the supplementary materials to give sufficient background context for the use of the self-report measure in this study. In response to the reviewer’s suggestion, we have now clarified the instructions given to the academic experts in the supplementary materials.*

The phrase in line 98f—“Attitudes towards gender pronouns categorisation (e.g., beliefs regarding traditional and preferred pronouns)”—does not sufficiently explain the construct being measured. A precise definition or detailed description is needed to specify the attitude object. For example, what exactly is meant by “gender pronouns categorisation”?

As you intend to present your study to an international audience, it would be helpful to explicitly state what is meant by “traditional” and “preferred” pronouns in English, as these terms may vary across languages.

*Response: In the introduction as requested, we have added clarification of the process we call gender pronoun categorization which we regard is the normative pattern of pronoun assignment that is typically used in various contexts. One such context is the assignment of gender pronouns based on physical appearance of an individual which is the focus of the current set of studies.  However, we have also acknowledged that these categories are not fixed, universal nor do they capture the full diversity that exists beyond our paradigm.*

Additionally, the abstract refers to “attitudes towards misgendering.” Are these the same attitudes or distinct constructs? Please clarify and, if they differ, define both explicitly and ensure consistent terminology throughout the manuscript.

*Response: We have amended this to refer to attitudes toward gender pronoun categorization.*

Looking at the items in Table 1 (Attitudes to gender pronouns), some appear to assess whether participants view pronouns as strictly binary (he/she) or accept non-binary options, others reflect attitudes toward the social norm of sharing pronouns, and others relate to respecting individual identity. Please note that even if a scale demonstrates internal reliability (i.e., consistent responses across items), its items can still cover multiple themes. This should be acknowledged and conceptually addressed.

*Response: Our EFA and CFA supported a one-factor solution with good fit, indicating that the items share substantial common variance and can be treated as a unidimensional scale. However, in line with the reviewer’s suggestion we recognise that whilst the item constructs are strongly correlated in our sample, they are conceptually distinguishable. We now clarify this in the manuscript and note that the total score reflects a broad attitudinal dimension encompassing these related components.*

In sum, the introduction should include a clear and comprehensive definition of the attitude construct. This definition could also incorporate the two sub-scales—difficulties in using gender pronouns and worries about using gender pronouns—as they seem to conceptually belong to the overarching attitude construct, even though they were empirically distinguished through factor analysis. Including this clarification would help readers understand how the sub-scales relate to the main construct and strengthen the conceptual framework of the measure.

*Response: We thank the reviewer for this helpful suggestion. We have revised the Introduction to provide a clearer conceptual definition of the self-report measures and their relationships. Specifically, we now clarify that overall attitudes toward gender pronouns were assessed using a validated attitudinal scale, while perceived difficulties using pronouns and worries about misgendering were assessed using separate self-report scales. We explain that difficulties reflect perceived cognitive or linguistic challenges in pronoun use, whereas worries capture affective concerns about using pronouns incorrectly and causing social harm. Although these constructs were empirically distinguishable, we note that they are conceptually related in that they reflect how individuals evaluate, engage with, and emotionally respond to changing norms around gender categorization. This clarification has been added to the Introduction to strengthen the conceptual framework and aid reader interpretation.*

The manuscript suggests both age-related changes (e.g., cognitive slowing and changes in inhibitory control) and cohort effects (e.g., differences in social norms across generations), with age-related changes linked to interference effects in the congruence tasks and cohort effects linked to the self-report measure. Currently, these perspectives appear in different parts of the manuscript. I recommend making this contrast more explicit, especially since testing age as both a continuous and categorical variable is informative but does not fully disentangle age-related changes from cohort effects. For example, this distinction could be highlighted when presenting the hypotheses to clarify which effects are expected to reflect aging and which reflect generational differences.

*Response: Thank you for this insightful comment. We agree that age can reflect both life-course (aging-related) variation and cohort differences in social experience. We have now clarified this conceptual distinction in the Introduction by noting that age may capture both aging-related and generational influences. We also added a brief explanation in the Methods describing why we examined both continuous and categorical age predictors, and we now explicitly state in the Results that generational analyses yielded the same pattern as the continuous-age models. Finally, we have revised the Discussion to acknowledge that, because the study is cross-sectional, aging and cohort effects cannot be fully disentangled, and that the convergence of continuous-age and generational analyses suggests that neither type of effect meaningfully altered our conclusions.*

I consider the mixed-effects modeling approach that includes faces as a random effect when testing Hypothesis 1 as superior, as this method avoids the loss of information caused by averaging across trials. Additionally, the table in the supplementary materials indicates a congruency-by-task interaction with p < .20. Based on Figure 1, this appears to suggest that the interference effect is slightly stronger in the orientation task. This interpretation would be consistent with the generally higher response times observed for the orientation-face task, which likely reflects greater overall task difficulty. While neither analytical approach provides support for Hypothesis 1, I suggest considering whether this small difference in findings might still be relevant to report for completeness.

*Response: We thank the reviewer for their thoughtful observation. We agree that the mixed-effects analysis retains more trial-level information and therefore included this supplementary exploratory analysis for completeness. However, as this analysis was not part of our preregistered plan, we are cautious about interpreting patterns that may arise by chance, particularly given the increased Type I error risk associated with unplanned analyses.*

*Although the congruency × task interaction in the trial-level model had a numerical value in an interesting direction, its p-value (p = 0.178) and confidence interval both clearly encompassed zero, indicating no statistical evidence for an interaction. Consistent with this, the effect size was extremely small. Importantly, the study was highly powered to detect such small effects, we had a large sample (N = 600) and over 110,000 trial-level observations. The absence of a significant interaction in this context therefore suggests that any difference between tasks is negligible.*

*For these reasons, and to avoid overinterpretation of exploratory findings that are not statistically robust, we have chosen not to highlight the p = 0.178 effect in the main text, but we continue to report the full model results in the supplementary materials for transparency.*

Minor:
Caution is warranted with reference to the findings for Hypothesis 4, as the interaction between Overall Attitudes to Gender Pronouns and Age seems to yield a p-value below .20. This result should be reported. Since the hypothesis predicted a null finding, note that the result is consistent with this expectation (as it does not meet convential significance criteria) but should not be interpreted as proof of the null.

*Response: We thank the reviewer for this helpful clarification. In our preregistered analysis for Hypothesis 3 (which we believe the reviewer is referring to, with results presented in Table 4), the Attitudes × Age interaction (p = 0.187) and all related predictors had confidence intervals that clearly included zero, indicating no statistically detectable association with interference scores. Because our hypothesis predicted no effect, these non-significant results are consistent with this expectation; however, we agree that they should not be interpreted as evidence for the null, and we do not treat p-values above .05 as meaningful. We have amended the Results section to state that none of the attitude predictors nor their interactions with age showed a statistically detectable association with interference scores (all ps for predictors > .18), without implying that the absence of significance constitutes proof of the null.*

When discussing the faster responses to the pronoun–face task compared to the orientation task (line 375ff), the explanation appears confusing, as faster responses are typically interpreted as indicating more automatic processing. I recommend clarifying this point and, additionally, considering other factors that may contribute to the observed difference. For example, task complexity—words in the orientation task are longer—and stimulus familiarity—participants may be more familiar with up–down photos than down–up photos. Including these considerations would provide a more comprehensive interpretation of the response time differences.

*Response: Thank you for highlighting this. We agree that our original interpretation required clarification. We have revised the discussion to provide a clearer and more accurate explanation of why responses were faster in the pronoun–face task. Specifically, we now note that faster responses likely reflect greater processing fluency, and we additionally acknowledge that task-related factors may also contribute to the observed differences in response times. This revision offers a more comprehensive and balanced interpretation, in line with the reviewer’s suggestion.*

Please ensure that all labels used in figures and tables are clearly explained in the captions or text and that they are consistent with the terminology used throughout the manuscript (for example, the label for the criterion used in the analysis presented in Table 4 - interference effect - should have been introduced when presenting the statistical analysis for the respective hypothesis (l. 257).

*Response: We have now amended the methods and results section for Hypothesis 3 to indicate that the interference effect is calculated as the difference in reaction times (ms) between incongruent and congruent trials.*

Ensure that the table headers for regression analyses clearly state the criterion and the predictors; for example the header in Table 4 would be easier to comprehend as “Linear regression models predicting interference effects in the pronoun–face task from self-reported attitudes toward gender pronouns (overall attitudes, perceived difficulties, and worries), in interaction with age (years)”.

*Response: We have amended the titles for all of the tables in line with the reviewer’s suggestion.*

Consider standardising the way hypotheses are presented. In academic writing, it is common to use numerals and to capitalise the term; for example: ‘Hypothesis 1’ rather than ‘hypothesis one.’ (also ‘Dataset 1’ rather than ‘dataset 1’)

*Response: We have amended this.*

Reviewer #2: Thanks for addressing all my comments and suggestions. I believe that with the current structure the paper is ready for publication

*Response: We sincerely thank the reviewer for their thoughtful engagement with our manuscript and for the helpful comments and suggestions provided throughout the review process. We are very grateful for the time and care taken in evaluating our work, and we are pleased that the revised manuscript is now considered ready for publication.*
